# The Administration of Ether

**Published:** 1895-09

**Authors:** John Freeman

**Affiliations:** Anæsthetist to the Bristol General Hospital and to the Home for Crippled Children


### THE ADMINISTRATION OF ETHER.

BY

JOHN FREEMAN, F.R.C.S. Ed.,

*Anæsthetist to the Bristol General Hospital and to the Home for Crippled Children.*

THE superiority of ether over chloroform as a general anæsthetic is now acknowledged by most anæsthetists, but opinions still differ as to the best method of administering it. The difficulties which have arisen in the production of ether narcosis have sometimes been so great, that discredit has been thrown upon this agent, and the more dangerous one—chloroform—has taken its place.

Believing that most of the difficulties associated with ether can be prevented, I am venturing to bring forward the method I have adopted in my last 400 cases, as I have found the results obtained by it are better than when I gave it in the usual way. The difference between the two consists in my using a more dilute vapour both in getting the patient under, and afterwards during the progress of the operation. I do not in this paper intend to discuss the employment of nitrous oxide as a preliminary to ether. No doubt this does remove from the patient the initial discomfort of ether, but it is a method which requires a considerable amount of practice to change from one anæsthetic to the other without the patient coming round, and moreover, it necessitates a more complicated apparatus. Although it is now close on twenty years since Clover first introduced this combination of anæsthetics, it does not appear to have been taken up to any great extent by general practitioners. By the method I am about to describe, patients can, in the great majority of cases, be anæsthetised quietly, and I believe without feeling any great discomfort, by ether alone. Many of them, if asked after they have come round what the sensation of going under was, will answer they remember nothing about it.

Clover's inhaler is the one I always use. This apparatus is so well known that I need not describe it, but there is one part of it I should like to draw attention to; viz., the air bag, which is often made too small. The size I like best is that supplied by Messrs. Down Bros. on their inhaler. This has a fluid capacity of 120 oz.

In using this inhaler, it is the custom of many to rotate the ether reservoir until the indicator is at the letter F (full on) to get the patient under, and then to let the indicator point to the figure 2 for the remainder of the operation. I used to employ this method; but now I find it is easy, in 90 per cent. of my cases, to get good anæsthesia by rotating the reservoir until the indicator reaches the figure 2 for getting the patient under, and after that the indicator can be kept midway between 1 and 0. Although this is less ether than is generally given, I wish it to be understood that the resulting anæsthesia is a complete one. The cornea is insensitive, the muscles are relaxed, and there is an absence of all such reflex phenomena as coughing, vomiting, &c. Though there are not the same dangerous reflex symptoms seen with incomplete ether anæsthesia as in the same stage when chloroform is the anæsthetic, yet this condition should always be avoided. It is somewhat dangerous to the patient, as the vomiting or spasm of glottis may cause asphyxial trouble; and struggling or rigidity of muscles interferes with the proper performance of the operation. In fact it is nearly always during incomplete anæsthesia that difficulties arise under ether.

Occasionally I meet with patients who require more ether than I have mentioned. These are nearly always strong muscular men, and frequently they have been alcoholics.

*Method of Administration.*—After 1½ oz. of ether have been poured into the reservoir, the indicator of which is at 0, the inhaler, without the air bag, is applied to the face. By telling the patient he is only having air he will breathe with more confidence, and soon get accustomed to the face-piece. After he has had five or six breaths in this way, the air bag can be fixed on to the inhaler. If the anæsthetist has not already blown out the bag, the face-piece must be raised at each inspiration, and lowered in time to catch the expiration ; in this way the patient fills the bag with air himself. As soon as the bag has become *thoroughly* distended, keep the face-piece applied closely and let the patient have about six breaths to and fro into the bag; then begin to turn the ether reservoir slowly, keeping the face-piece applied all the time.

It should take from a minute to a minute and a half for the indicator to reach the figure 1. Up to this time the patient will most likely have been breathing in a quiet, rather hesitating manner; but now he suddenly begins to take deep breaths, showing that his air-passages have got accustomed to the ether, and he generally loses consciousness at this stage. Now the reservoir can be rotated rather more quickly until the indicator points to the figure 2, where it can be left in the great majority of cases: of course if the patient does not go under with this, more ether must be turned on; but as I have already said, I find this sufficient in 90 per cent. of my cases. The time required to get a patient under from the moment I commence to turn on the ether varies from two and a half to four minutes. The indicator is kept at 2 for five minutes, so as to get the patient well under the influence of the ether. It is then turned back to 1 for another five minutes, and after that it can be kept midway between 1 and 0 until the operation is finished.

Now another very important factor in the production of ether anæsthesia has to be considered; viz., the air supply. During the time the patient is going under, it is essential that the air supply be limited to a considerable extent. It is by allowing too much air at this stage that most of the difficulties connected with ether are brought about. As a rule it is not necessary to allow any fresh air from the time the ether is first turned on until the breathing begins to get stertorous—a period of from two to three minutes—but for this result to be obtained, the bag must have been quite full of air when the ether was begun, and the larger size bag has a great advantage over the smaller one.

Directly the breathing begins to get stertorous, showing that the patient is nearly under, one breath of air may be given; but if many are given, the patient rapidly begins to come round. One breath will be sufficient to carry him on until the corneal reflex is lost. By this time his face will have a flushed appearance, and it may be a little dusky; but this need cause no anxiety, for now a few breaths of fresh air may be given until the face regains its normal colour. After complete narcosis has been induced, the anæsthetist must allow enough air to prevent any cyanosis, and on the other hand he must limit it sufficiently to prevent the patient coming out of the anæsthesia. Since patients vary so much under anæsthetics, it is almost impossible to lay down any rule with regard to the amount of air to allow. Not only is there a great difference between different patients, but the same patient varies according to the length of time he is under the anæsthetic. For instance, the quantity of fresh air that should be given to the patient after he has been under the anæsthetic for, say, an hour would be quite incompatible with good anæsthesia in an early stage of the administration. Generally speaking, one breath in eight or ten is all that can be allowed for the first twenty minutes, after that, one in every five or six may be given ; but all kinds of variations will be met with, and the only real guides are the degree of anæsthesia and the colour of the patient's face. So long as there is no cyanosis we may rest assured that the patient is being allowed sufficient air, and by using a less concentrated vapour of ether there is less tendency to duskiness than when a strong one is given.

I think it is best to give the ether slowly at the beginning of the administration, because by this means the patient's mind becomes dazed before the vapour is sufficiently strong to cause discomfort. When a strong vapour is commenced with, it may bring about spasm of the glottis, which produces that horrible sensation of suffocation of which patients complain. In the hands of experts this method may be employed satisfactorily; but when I have seen it attempted by beginners it has frequently been the cause of great trouble—producing holding of breath, coughing and retching, and where the patient has been a powerful muscular one it has ended in complete failure.

There are one or two interesting points connected with this method of maintaining anæsthesia. Generally there is a less copious secretion of saliva and mucus. This is perhaps one reason why there is less cyanosis. The air passages are not irritated so much as by the stronger vapour, and so that excessive accumulation of mucus in the trachea, which sometimes causes trouble, is less likely to take place. Also, the pupil is a smaller one (about 2½ mm.): this does not mean that the patient is not under sufficiently, for there is an absence of all signs of incomplete anæsthesia; neither is it due to the asphyxial element having been carried too far, for during the early part of the administration, when the indicator is at 2 and the air-supply limited, the pupil is moderately dilated. The contraction occurs later on, when the indicator is turned back and the patient is having a good supply of air as shown by the normal colour of the face.

As far as I have been able to observe, the after-discomfort of the patient is less with this method. He comes round more quickly, there is less vomiting, and that disagreeable taste and smell of ether which some patients complain of so much passes off sooner. I think also this weaker vapour is less likely to produce after-affections of the air passages. When administered in this manner, ether can be safely given to children and to old people.

